# Discovery of a Metabolic Signature Predisposing High Risk Patients with Mild Cognitive Impairment to Converting to Alzheimer’s Disease

**DOI:** 10.3390/ijms222010903

**Published:** 2021-10-09

**Authors:** Yi-Long Huang, Chao-Hsiung Lin, Tsung-Hsien Tsai, Chen-Hua Huang, Jie-Ling Li, Liang-Kung Chen, Chun-Hsien Li, Ting-Fen Tsai, Pei-Ning Wang

**Affiliations:** 1 Aging and Health Research Center, National Yang Ming Chiao Tung University, Taipei 112, Taiwan; yilonghuang@nycu.edu.tw (Y.-L.H.); chaohsiunglin@nycu.edu.tw (C.-H.L.); l30840143@nycu.edu.tw (J.-L.L.); lkchen2@vghtpe.gov.tw (L.-K.C.); 2 Department of Life Sciences and Institute of Genome Sciences, National Yang Ming Chiao Tung University, Taipei 112, Taiwan; denny1210.y@nycu.edu.tw; 3 Advanced Tech BU, Acer Inc., New Taipei City 221, Taiwan; Vincent.Tsai@acer.com (T.-H.T.); Zack.Li@acer.com (C.-H.L.); 4 Center for Geriatrics and Gerontology, Taipei Veterans General Hospital, Taipei 112, Taiwan; 5 Taipei Municipal Gan-Dau Hospital, Taipei 112, Taiwan; 6 Institute of Molecular and Genomic Medicine, National Health Research Institutes, Zhunan 350, Taiwan; 7 Division of General Neurology, Department of Neurological Institute, Taipei Veterans General Hospital, Taipei 112, Taiwan; 8 Department of Neurology, School of Medicine, National Yang Ming Chiao Tung University, Taipei 112, Taiwan

**Keywords:** Alzheimer’s disease, mild cognitive impairment, untargeted metabolomics, plasma, feature selection

## Abstract

Assessing dementia conversion in patients with mild cognitive impairment (MCI) remains challenging owing to pathological heterogeneity. While many MCI patients ultimately proceed to Alzheimer’s disease (AD), a subset of patients remain stable for various times. Our aim was to characterize the plasma metabolites of nineteen MCI patients proceeding to AD (P-MCI) and twenty-nine stable MCI (S-MCI) patients by untargeted metabolomics profiling. Alterations in the plasma metabolites between the P-MCI and S-MCI groups, as well as between the P-MCI and AD groups, were compared over the observation period. With the help of machine learning-based stratification, a 20-metabolite signature panel was identified that was associated with the presence and progression of AD. Furthermore, when the metabolic signature panel was used for classification of the three patient groups, this gave an accuracy of 73.5% using the panel. Moreover, when specifically classifying the P-MCI and S-MCI subjects, a fivefold cross-validation accuracy of 80.3% was obtained using the random forest model. Importantly, indole-3-propionic acid, a bacteria-generated metabolite from tryptophan, was identified as a predictor of AD progression, suggesting a role for gut microbiota in AD pathophysiology. Our study establishes a metabolite panel to assist in the stratification of MCI patients and to predict conversion to AD.

## 1. Introduction

Alzheimer’s disease (AD) is currently the most common dementia disease worldwide, and it has been estimated that it will affect more than 100 million people by 2050 [1]. Up to the present, clinical trials testing AD disease-modifying therapies have failed to provide any promising solutions [2]. In this context, two new directions for AD therapeutic development have been proposed. Firstly, that possible pathological mechanisms other than the amyloid-driven hypothesis should be explored and, secondly, that individuals during the early stages of AD should be identified, with this being the time frame in which intervention is most likely to be possible. With respect to AD diagnosis, mild cognitive impairment (MCI) is often considered to be the prodromal stage of clinically diagnosed AD. Although MCI patients are at high risk of developing AD, they are pathologically heterogeneous and seem to consist of a range of different subtypes [3]. For example, not all MCI patients progressed to dementia and some remained stable for a relatively long period of time, while a small portion even reverted to normal cognition [4,5]. More importantly, some MCI patients do not develop AD, but rather develop other types of dementia [6]. Therefore, predicting the individual development pattern of a given patient at the MCI stage is critical when physicians are diagnosing AD; nevertheless, this remains an unmet clinical need.

Current clinical methods for diagnosing AD include brain imaging and the measurement of β-amyloid and tau levels in the cerebrospinal fluid [7]. Considering the high cost and invasive nature of these approaches, the development of alternative diagnostic tools would be very useful when carrying out early AD detection [8,9]. Establishing one or more blood-based metabolic signatures would be a rapid and less invasive approach to assisting AD diagnosis [10]. Although over 200 metabolites have been reported to be potential AD biomarkers in plasma/serum [11,12,13,14,15,16], only a few reports have reported metabolites that are closely linked to AD conversion among MCI patients [15,17,18]; furthermore, most of these metabolites are lipids owing to the use of a commercial kit.

In the present study, we have collected untargeted metabolomic data from human plasma samples to allow a comprehensive investigation of the metabolic alterations that have occurred in a range of MCI patients. Univariate, multivariate statistical analysis, and machine learning (ML) models were used for MCI stratification based on clinical diagnosis at follow-up. The ultimate goal of this study is to establish a metabolic signature panel that will identify the various MCI subtypes, and thus provide for individualized treatment of patients during AD development.

## 2. Results

### 2.1. Demographics of the MCI Patients and Metabolomic Analysis in Plasma Samples

A total of 61 human plasma samples, collected under IRB regulations, were analyzed during the present study; these included 48 patients diagnosed as MCI. To investigate the association of the plasma metabolites with AD conversion, we focused on the longitudinal data collected from the MCI patients who proceeded to AD (P-MCI subjects, *n* = 19); these are the patients that underwent MCI-to-AD conversion during the observation period. For comparison purposes, the MCI patients who remained MCI or exhibited an alleviation of symptoms over the 3- to 5-year study period were defined as stable MCI (S-MCI, *n* = 29). The demographics of these subjects are shown in Table 1, including age, gender ratio, education, and cognition evaluation. Age, baseline MMSE, and APOE ε4 alleles were significantly associated with AD progression risk (all *p* < 0.05). Metabolomics analysis of these plasma samples was carried out using a liquid chromatography-mass spectrometer (LC-MS). To circumvent analytical drift among the batches, a pooled quality control (QC) for normalization was used, as suggested previously [19]. Upon normalization, a total of 3749 common features were selected for comparison across all samples.

### 2.2. Metabolic Alterations in Patients Who Have Progressed from MCI to AD or Who Have Remained MCI during the Observation Period

**Figure 1:**
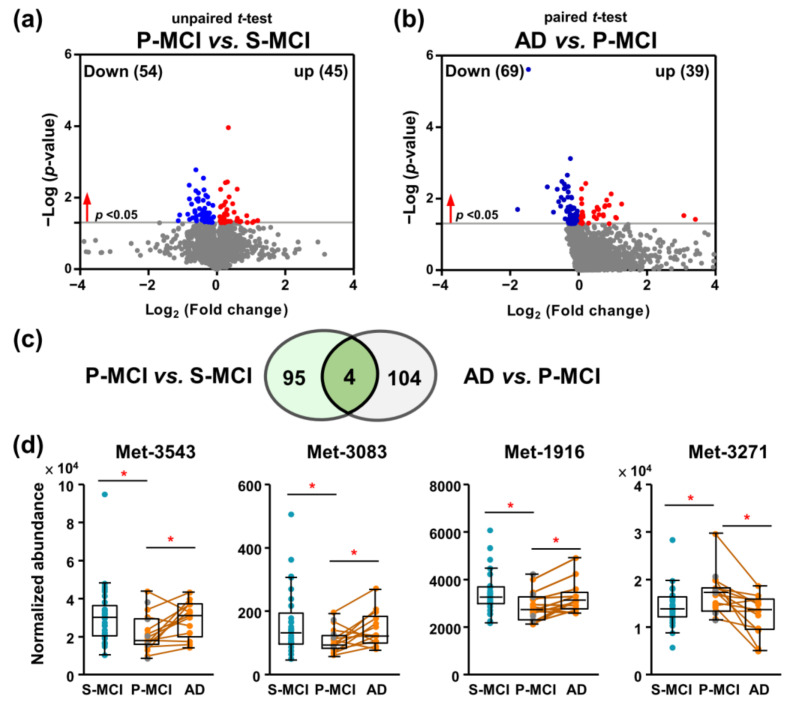
Longitudinal and cross-sectional analysis comparing the plasma metabolomic data of MCI patients. (**a**) Comparison of the metabolic profiles between 19 P-MCI and 29 S-MCI patients based on independent *t*-tests. A total of 99 metabolic features were statistically significant (*p* < 0.05). (**b**) Comparison between 13 paired P-MCI and AD groups to identify differential metabolites associated with AD development based on paired t-tests. A total of 108 metabolic features were statistically significant (*p* < 0.05). (**c**) A Venn diagram of the comparison between the P-MCI vs. S-MCI and P-MCI vs. AD metabolic features. (**d**) Box and whisker plots of four common metabolic for differentiating the S-MCI, P-MCI, and AD patients based on paired and independent *t*-tests. Paired samples are connected by lines. Grey dots represent six P-MCI subjects without AD metabolomics data. * *p* < 0.05. S-MCI, stable MCI; P-MCI, MCI proceeding to AD.

Firstly, we investigated whether these subgroups can be discriminated by univariate statistical analysis. Independent sample t-tests were used to compare the P-MCI and S-MCI groups, and a total of 99 significant (*p* < 0.05) differentially expressed metabolites were defined (Figure 1a). In addition, to investigate the association of the plasma metabolites with the AD transition, metabolic features from samples collected in the MCI stage of P-MCI patients were compared to those collected in the AD stage of same P-MCI patients. The paired t-test statistics upon AD transition of 13 P-MCI patients provided a total of 108 metabolic features with *p* values <0.05 (Figure 1b). The number of overlapping features among these two lists was limited (Figure 1c). Unexpectedly, all these metabolites did not continuously decrease or increase; for example, the levels of Met-1916 decreased in the P-MCI group compared with the S-MCI group, whereas the same metabolite increased on MCI-to-AD progression, as shown in Figure 1d. These analyses indicated that the plasma metabolome changes across the pathology of AD were non-linear. This prompted us to use ML methods to facilitate the task of classification.

### 2.3. Building Machine Learning-Based Models for the Classification of AD, P-MCI, and S-MCI

**Figure 2:**
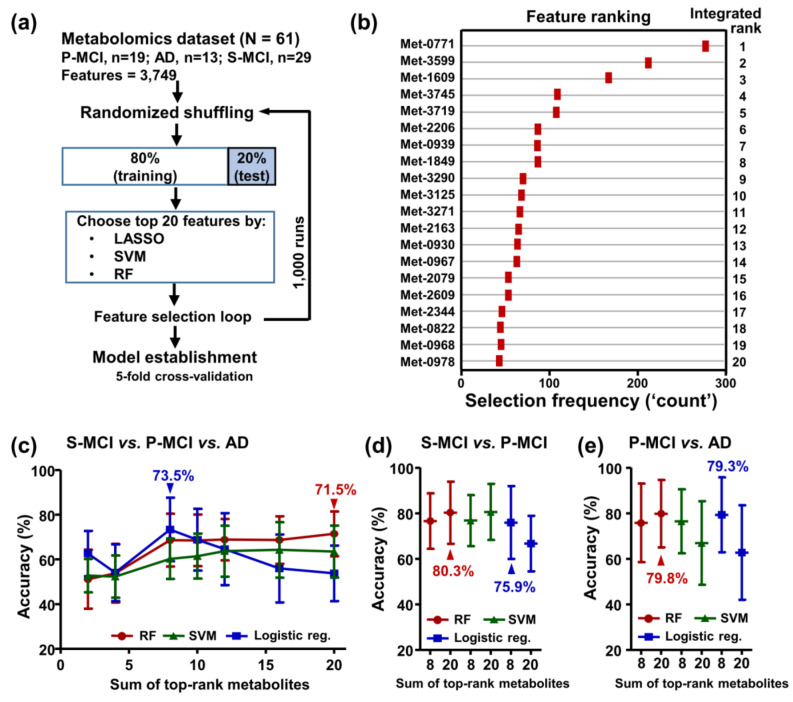
Model development and evaluation for biomarker discovery involving the risk assessment of AD conversion. (**a**) The workflow of the feature selection used to establish a signature panel for differentiating the S-MCI, P-MCI, and AD patients. In each loop, the dataset was partitioned into a ‘Training Set’ and a ‘Test Set’ and the top 20 most informative features were selected as predictors. The predictive performance of the classifiers was estimated by fivefold cross-validation. (**b**) The top 20 differentiating features ranked by a combination of the Lasso, RF, and SVM algorithms. (**c**) Accuracies of classification among the S-MCI, P-MCI, and AD patients using a varying number (2–20) of the top-ranked features based on the RF, SVM, and logistic regression models. (**d**) Prediction accuracies of binary classification models during which all MCI patients were assigned into either the P-MCI group or the S-MCI group. (**e**) Accuracies of the binary classification models to predict MCI-to-AD progression. The accuracies (shown as mean ± standard deviation) were determined using fivefold cross-validations. S-MCI, stable MCI; P-MCI, MCI proceeding to AD.

LASSO, support vector machine (SVM), and random forest (RF) were used to identify a subset of features that are most relevant to classifying the S-MCI, P-MCI, and AD samples from the original 3749 features. Subsequently, fivefold cross-validation with ten repetitions was utilized to eliminate bias and improve the reliability of the results. A schematic diagram illustrating the overall procedure is shown in Figure 2a. The top 20 features with high selection frequencies during the ML-based group classification are shown in Figure 2b. Based on the performance of three-group classifications obtained using popular ML algorithms including logistic regression, RF, and SVM, we chose the top-ranked eight features trained using the logistic regression model (Figure 2c). Using these eight features, we were able to classify the selected test subjects into the S-MCI, P-MCI, and AD groups at an average classification accuracy of 73.5 ± 14.2%. Alternatively, to focus on the classification between P-MCI and S-MCI subjects, better values were obtained for accuracy (80.3 ± 13.6%) using the RF model (Figure 2d). Similarly, a comparable accuracy value (79.8 ± 14.8%) was observed for the P-MCI–AD comparison (Figure 2e). On the other hand, the classification model training using the 20 metabolites with most significant association resulted in a modest predictive ability (accuracy = 49.8–65%, Figure S1), which suggests that the statistical significance of the univariate analysis did not produce an optimized marker combination.

### 2.4. Multivariate Statistical Analysis of All Three Groups

**Figure 3:**
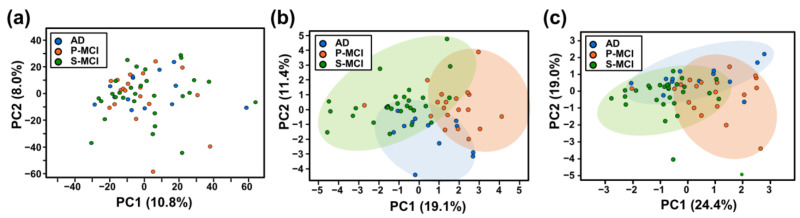
Principal component analysis (PCA) was used to derive the major distribution patterns from metabolomics data for all three groups. (**a**) PCA of 3749 metabolic features from 61 samples of S-MCI, P-MCI, and AD patients. (**b**,**c**) PCA representation of the twenty (**b**) or eight (**c**) metabolites selected by the machine learning algorithms. S-MCI, stable MCI; P-MCI, MCI proceeding to AD.

Next, we applied principal component analysis (PCA), which is an unsupervised method, to uncover the potential distinction across the three classes (29 S-MCI, 19 P-MCI, and 13 AD). The P-MCI, S-MCI, and AD groups share a very similar distribution based on the original 3749 metabolites (Figure 3a). Although the most frequently selected features are better able to discriminate the three cognitively impaired populations, a subset of each group of samples still overlapped among these groups using the PCA model (Figure 3b,c). Thus, currently, differentiation between the three groups of cognitively impaired patients using conventional statistical analysis remains a challenge.

### 2.5. Development of an Eight-Metabolite Index for Binary Discrimination

**Figure 4:**
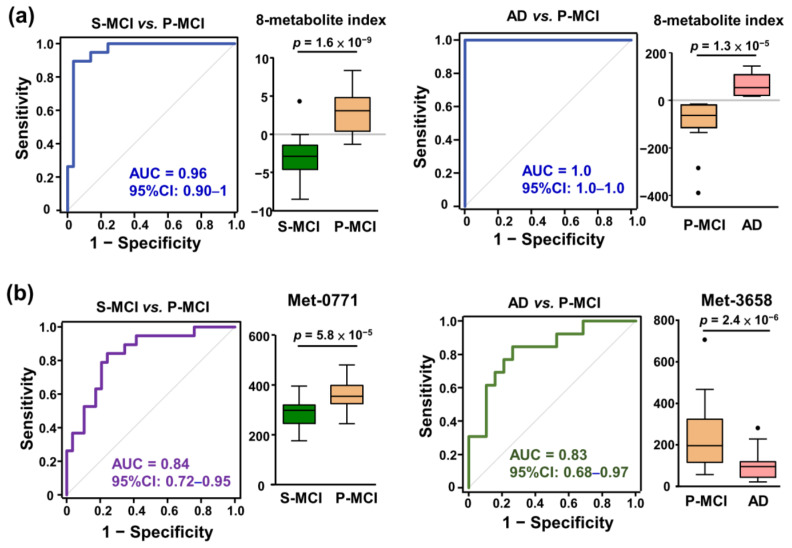
ROC curve analysis assessing the performance of the binary diagnostic tests using the various statistical and machine learning strategies. (**a**) ROC curves based on eight-metabolite index generated using the logistic regression model to discriminate between P-MCI vs. S-MCI groups and AD vs. P-MCI groups. The associated AUC and 95% confidence interval (CI) are indicated. (**b**) ROC curves based on univariate statistical significance to discriminate between P-MCI and S-MCI groups and the AD and P-MCI groups. The grey diagonal line represents random classifier performance (AUC = 0.5). Each metabolic feature and metabolite index is depicted in a vertical box plot where the horizontal lines represent the median value and the black dots represent the outliers (>1.5 interquartile range from ends of the box). Student’s *t*-test was used to assess the significance of the difference between two groups. AUC, area under the ROC curve. S-MCI, stable MCI; P-MCI, MCI proceeding to AD; ROC, receiver operating characteristic.

Finally, a plasma eight-metabolite index was created by combining eight metabolite abundances using the coefficient of each metabolic feature in the specific binary logistic regression models. In the S-MCI–P-MCI classifier system, the eight-metabolite index significantly differentiated between the P-MCI and S-MCI groups, and the area under the receiver operating characteristic (ROC) curve was 0.96 (95%CI: 0.9–1; Figure 4a left panel). Additionally, ROC analysis showed perfect classification (AUC = 1) for subjects in the P-MCI and AD groups (Figure 4a right panel). Both AUC values were higher than those obtained from the statistically significant metabolites between (1) P-MCI and S-MCI subjects and (2) P-MCI and AD subjects (Figure 4b). Altogether, our findings indicate that a higher predictive performance can be obtained by integrating the metabolomic signature panel suggested by ML.

### 2.6. Identification of the Metabolites That Seem to Reflect the Risk of AD Conversion

**Figure 5:**
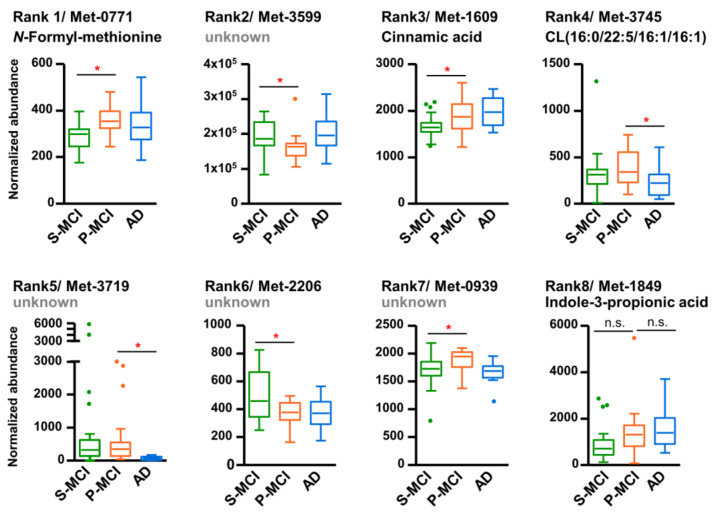
Box and whisker plots of eight representative features for differentiating the S-MCI, P-MCI, and AD groups. The box defines the 25th and 75th percentile of the intensity distribution and the horizontal line within the box representing the 50th percentile (median). Outliers are defined as having an intensity exceeding 1.5 interquartile ranges lower than 25th percentile or higher than the 75th percentile, which were indicated as dots. * *p* < 0.05. S-MCI, stable MCI; P-MCI, MCI proceeding to AD; CL, cardiolipin; n.s., non-significant.

Among top twenty most predictive features for AD risk assessment, nine metabolites were structurally assigned based on their accurate mass and fragmentation pattern; these metabolite identities are summarized in Table 2. Expression profiles across the P-MCI, S-MCI, and AD groups of these top eight metabolites, which were the most frequently selected, are shown in Figure 5. The majority of the metabolites, specifically five out of eight, were found to be significant altered in the P-MCI group compared with the S-MCI group, whereas no significant changes were observed when the P-MCI to AD groups were compared. Of note, the rank 1 metabolite, *N*-formylmethionine, was also the metabolic feature exhibiting the most statistical significance (smallest *p*-value) within the S-MCI versus P-MCI comparison (Figure 4b). Two other metabolites showed a significant reduction on the MCI-to-AD transition. However, these were found not to differ significantly between the P-MCI and S-MCI groups. Interestingly, although no significant inter-group variation was detected, the overall increasing trend of indole-3-propionic acid from S-MCI to AD suggested a potential link for cognitive impairment (rank 8 in Figure 5).

## 3. Discussion

### 3.1. Plasma Biomarkers That Can Be Used to Diagnose AD and Track Its Progression

It has been shown that the AD-related disturbances of serum/plasma metabolome are highly variable with low reproducibility [20,21]. Importantly, metabolic alterations associated with AD may not be able to differentiate patients that have distinct MCI stages in terms of their progression rate and direction. It is, therefore, important to identify the unique signatures associated with different MCI patients and explore how these vary in terms of their future development towards either AD or non-AD dementia. However, there is currently no clinical method able to accurately predict which MCI subjects will later progress to AD. In the present study, we have focused on establishing a robust prediction model for evaluating AD risk based on the patient’s metabolite biomarker signature.

### 3.2. Marker Panel Separating P-MCI and S-MCI Patients

Owing to the complexity of MCI pathology, a single biomarker is unlikely to yield enough sensitivity and specificity, and thus multiple biomarkers are likely to be necessary to facilitate disease monitoring. In this study, the differential metabolites observed in P-MCI patients did not deteriorate further during MCI-to-AD progression (Figure 1), which indicates that relationship between cognitive impairment and the plasma metabolome appears to be complex. In line with this observation, conflicting direction for the associations between AD progression risk and AD diagnosis in CSF samples have been reported [22]. A recent study also excludes a “linear” pathology from cognitively normal to AD [23]. Altogether, taking into account the long preclinical phase of dementia, a variety of dementia-associated metabolic alterations are likely to be presented during the MCI stage. Thus, there seems to be an exciting opportunity for using multivariate metabolomic markers to classify MCI patients at high risk of converting to AD.

Considering the high-dimensional context, as well as the high degree of metabolite-metabolite interaction, found in untargeted metabolomics data [24], logistic regression, together with feature selection algorithms such as LASSO, RF, and SVM, are now being increasingly applied to the analysis of metabolomics data [25,26]. Here, the interaction effects between 3749 metabolic features were taken into account by including RF, LASSO, and SVM as selection algorithms in order to establish a minimal metabolic signature panel set (Figure 2a). Furthermore, the eight top-selected metabolic features, when combined, were able to differentiate stable MCI subjects, MCI subjects who later converted to AD, and AD patients, with an overall average accuracy of 73.5%. PCA also showed moderate separation among three classes (S-MCI, P-MCI, and AD) when the top-selected metabolic features were used (Figure 3).

Notably, the best performing model was derived from the binary class discriminations of S-MCI–P-MCI (80.3% accuracy) models (Figure 2d). Several machine learning-based approaches have been proposed for predicting MCI-to-AD progression [27,28], but most of them incorporated cognitive/functional markers and brain magnetic resonance imaging (MRI). Very little data are available on the accuracy of discriminating P-MCI from S-MCI using only blood metabolome data, and only one report has shown comparable accuracy (72%) when predicting AD progression; this was based on the concentrations of three metabolites in MCI patients [17].

On the other hand, all of the metabolomics analyses carried out during this study would seem to be associated with short-term MCI-to-AD conversion, as defined by a previous study [27]. It will thus be interesting to further investigate whether the conversion signature panel proposed in this study exhibits a time window correlation; this would require the long-term follow-up of a future cohort. Meanwhile, the predictive ability of the present eight metabolite signature when identifying MCI patients with a risk of rapid progression to AD is highly important if one is carrying out clinical evaluation of a MCI patient and then wishing to intervene.

### 3.3. The Metabolite Index Can Be Used for Individualized Risk Prediction

Alterations in circulating metabolites, including lipids, amino acids, and hormones, have been associated with a risk of dementia [29]. To the best of our knowledge, there is no AD progression risk score/index available based on plasma metabolomics data in the literature. Therefore, we have developed a plasma eight-metabolite index, which is calculated using the coefficients in the P-MCI versus S-MCI logistic regression model. The eight-metabolite index is able to achieve an AUC of 0.96 in the corresponding ROC curve (Figure 4), which confirms it as having potential diagnostic utility for MCI subjects. The validity of the index needs to be further confirmed by examining diverse MCI subjects in order to rule out over-fitting of the logistic regression model owing to the limited sample size of the current study.

### 3.4. Potential Pathways Underlying the Risk for Progression to Dementia

In this work, we identified a panel of 20 metabolic features, which exhibits predictive potential for MCI subtypes (Table 2). These include amino acid derivatives (furosine, *N*-formylmethionine, and arginine), cardiolipin 16:0/22:5/16:1/16:1 and its precursor CDP-DG a-25:0/i-24:0, diet-related metabolites (cinnamic acid, citbismine F, and pyrogallol-1-sulfate), and gut microbial metabolite (indole-3-propionic acid). While a change in amino acid derivatives is frequently associated with diverse diseases, alteration of cardiolipin, a mitochondria-membrane phospholipid, in brain tissues was associated with AD and other neurodegenerative diseases [30,31]. Despite that cardiolipin was decreased in the AD mouse model, the causal relationship between cardiolipin and AD pathology was not confirmed. However, our results suggest that the circulating level of cardiolipin is important to stratify MCI patients with different AD risk. On the other hand, cinnamic acid was previously shown to stimulate lysosomal biogenesis and reduce amyloid plaque pathology in the AD mouse model [32]. It is suggested to further investigate whether plasma levels of cinnamic acid are associated with AD risk. In addition, *N*-formylmethionine is an initiator of protein synthesis in bacteria or mitochondria. Nevertheless, linkage of this mitochondria-derived metabolite to AD progression in MCI patients requires further investigation.

### 3.5. Implication of Identification of Gut Microbiota-Derived Metabolites

In our results, the metabolite, indole-3-propionic acid, an indole derivative converted from tryptophan by gut bacteria, was identified as a predictor of AD progression (Table 2). It has been reported that MCI and AD patients have altered gut microbiome compared with controls [33,34]. Previous research has suggested that gut diversity might impact the bioavailability of tryptophan and its downstream metabolites [35]. Whether this explains the association of indole-3-propionic acid with AD progression risk, specifically that there is only disturbance in P-MCI subjects, but not in S-MCI subjects, remains to be explored. In addition, pyrogallol-O-sulfate, a colonic microbiota-derived polyphenol catabolite, has been shown to improve cellular responses to oxidative injuries, and is able to pass through the blood–brain barrier in the cell model [36]. Therefore, the plasma pyrogallol-O-sulfate may directly affect brain function and is associated with AD risk in MCI patients. Given that accumulating evidence suggests a significant relationship between diet and gut microbiome [37], our results highlight the importance of studying the microbiome-dependent effects in MCI patients.

### 3.6. Conclusions

The current challenge when carrying out MCI stratification is a lack of reliable indicators at the molecular level. To address this issue, we have attempted to apply ML algorithms to screen suitable metabolites that are able to create a signature panel for MCI development. Our findings demonstrate that a combination of a high-dimensional (>3500 features) untargeted metabolomics dataset with ML algorithms allows an evaluation that assists in the stratification of diverse MCI patients. Such an assessment using metabolite signature panels should help determine the degree of cognitive impairment of a given MCI patient, as well as the risk of disease development for that MCI patient; both of the above are important in terms of early intervention, effective monitoring, and predementia management.

## 4. Materials and Methods

### 4.1. Study Subjects

The Institutional Review Board (IRB) of Taipei Veterans General Hospital approved the study protocol. MCI samples and AD samples were obtained from Taipei Veterans General Hospital with the consent of the patients; these included 48 patients who were later defined as P-MCI or S-MCI based on their progression time window (Table 1). Clinical diagnosis of AD and MCI was based on a physical examination, a clinical interview, and a neuropsychological assessment as recommended by the National Institute on Aging/Alzheimer’s Association workgroups in 2011 [38].

### 4.2. Sample Collection and Metabolite Extraction

Fasting blood was drawn using EDTA-coated vacuum tubes and centrifuged at 4 °C and 3000× *g* for 10 min in order to obtain plasma samples; these were then stored at −80 °C until analysis. Each plasma sample (40 μL) was spiked with two internal standards (1 ppm lysine-^13^C_6_, 1 ppm stearic acid-^13^C_18_) and deproteinized by the addition of 160 μL 100% methanol. After centrifugation at 4 °C and 13,000× *g* for 10 min, the supernatant was lyophilized and re-dissolved in ultrapure water for LC-MS analysis.

### 4.3. LC-MS Analysis

LC-MS analysis was carried out on a Waters Xevo G2-S Q-Tof tandem mass spectrometer coupled with Acquity UPLC (Waters) using a BEH C18 column (2.1 *×* 100 mm 1.7 μm, Waters) kept at 40 °C. The total run time was 9 min and was performed at a flow rate of 0.3 mL/min. The gradient conditions for sample analysis were 1% B from 0 min to 0.5 min, 1–100% B over 0.5 min to 4 min, and then the composition was held at 100% B for 1 min, which was followed by a return to the initial composition over 1 min; the final conditions were maintained for a further 3 min. The mobile phase A was an aqueous solution containing 0.1% ammonium hydroxide and mobile phase B was acetonitrile containing 0.1% ammonium hydroxide. Leucine enkephalin ([M − H]^−^ = 554.2615 m/z) was used for the continuous mass calibration. MS^E^ data were acquired at both low and high collision energy with a mass scan range of 50–1200 m/z. In addition to the above, a pooled quality control (pQC) reference was prepared by mixing 10 μL from each 61 plasma samples; this was then aliquoted and analyzed during each LC-MS analysis batch in order to provide a basis for normalization between the different LC-MS analysis batches.

### 4.4. Untargeted Metabolomics and Statistical Analysis

MS^E^ data in the negative mode were processed using Progenesis QI (Nonlinear Dynamics), which generated a collection of chemical features; these were represented by their retention time, mass-to-charge (m/z), and ion intensity. Triplicate measurements of each individual sample were averaged. Subsequently, the ion intensities of all the chemical features from the different batches were normalized against the pQC reference sample. The principle component analysis (PCA) of the selected groups was carried out using EZinfo (Umetrics, version 3.0.3). The detailed procedures for the pQC normalization are described in the Supplementary Methods. The ROC curve analysis was carried out using SPSS version 24.0 (SPSS Inc., Chicago, IL, USA).

### 4.5. ML-Based Classification of the S-MCI, P-MCI, and AD Groups

Firstly, the dataset was randomly split into an 80% training set and a 20% test set. We used the LASSO [39] algorithm to select the features for group classification, which was followed by analysis by two algorithms, SVM [40] and RF [41]; these were used to rank the metabolites by their coefficients and Gini importance values, respectively. To increase the reliability of the feature selection and ranking, the above processes were repeated 1000 times, and then the top-ranked features were selected and used to build a signature panel. A program was developed using R version 3.5.2 (https://www.r-project.org (accessed on 12 February 2021)), which allowed feature selection using the metabolomics data. Additionally, logistic regression, SVM, and RF models were trained based on the most frequently selected metabolites. The predictive accuracies of various models were evaluated by fivefold cross-validation with ten repetitions using Weka software version 3.8.2 (https://www.cs.waikato.ac.nz/ml/weka/ (accessed on 8 April 2021)), and in this way, the optimal (most accurate) number of metabolic features was identified.

### 4.6. Metabolite Annotation

Tentative identification of ionic features was made by matching monoisotopic mass (m/z) to the values held in the human metabolome database (HMDB) (http://www.hmdb.ca/ (accessed on 21 January 2021)) with a mass tolerance of 30 ppm. The most probable annotations were made by matching the fragmentation pattern against the in silico fragmentation database MetFrag built in Progenesis QI [42] with a mass tolerance of 50 ppm.

## Figures and Tables

**Table 1:** Characteristics of the 49 individuals defined as P-MCI patients and S-MCI patients.

Characteristic	S-MCI	P-MCI
Subject (*n*)	29	19
Age (years)	68.0 ± 8.5	76.0 ± 9.9 *
Female (%)	51.7	52.6
Education (years)	12.3 ± 3.8	10.9 ± 4.5
Baseline MMSE score	28.2 ± 1.8	25.2 ± 1.6 *
Conversion to AD dementia (MMSE score at follow-up)	No (27.3 ± 1.7)	Yes (22.6 ± 1.9 ^a^)
AD medication, *n* (%)	1 (3.4)	5 (19)
Aricept, *n*	1	4
Exelon, *n*	0	1
APOE ε4 carrier (%)	13.8	47.3 *
Follow-up (years)	5.5 ± 1.2	4.4 ± 1.6

Means ± standard deviation (SD) are shown for continuous variables; MMSE, Mini-Mental State Examination; APOE, apolipoprotein E. * *p* < 0.05. ^a^ Calculated from 13 patients.

**Table 2:** List of the nine ML-selected MCI-differentiating features with their identified metabolite annotation.

Rank	Description	Metabolic Feature	Compound ID	Adduct	Formula	Score	Fragment Score	Mass Error (ppm)
1	N-Formyl-L-methionine	Met-0771	HMDB0001015	M-H	C_6_H_11_NO_3_S	39.7	18.2	3.16
3	Cinnamic acid	Met-1609	HMDB0000567	M-H	C_9_H_8_O_2_	28.3	0.09	7.35
4	CL(16:0/22:5/16:1/16:1)	Met-3745	HMDB0056838	M-2H	C_79_H_140_O_17_P_2_	28.1	3.06	−2.31
8	Indole-3-propionic acid	Met-1849	HMDB0002302	M-H	C_11_H_11_NO_2_	38.5	0	−2.52
9	CDP-DG(a-25:0/i-24:0)	Met-3290	HMDB0116150	M-H	C_61_H_115_N_3_O_15_P_2_	38.5	20.6	18.06
12	Citbismine F	Met-2163	HMDB0034473	M-H	C_36_H_34_N_2_O_10_	33.9	0.03	17.64
13	Pyrogallol-1-O-sulphate	Met-0930	HMDB0060016	M-H	C_6_H_6_O_6_S	37	0	−6.93
14	L-Furosine	Met-0967	HMDB0029390	M-H	C_12_H_18_N_2_O_4_	35	0	−16.06
20	L-Arginine	Met-0978	HMDB0000517	M-H	C_6_H_14_N_4_O_2_	45.2	40.6	−9.27

## Data Availability

The data that support the findings of this study are available from the corresponding author upon reasonable request.

## Supplementary Materials

The following are available online at https://www.mdpi.com/article/10.3390/ijms222010903/s1.
