# Supplementary material for: Discovery of a Metabolic Signature Predisposing High Risk Patients with Mild Cognitive Impairment to Converting to Alzheimer’s Disease
Source: Int J Mol Sci. 2021 Oct 9;22(20):10903. doi: 10.3390/ijms222010903 (PMC8535253; doi:10.3390/ijms222010903)
Supplement: Supplementary file 1 [file ijms-22-10903-s001.zip › ijms-1402652-supplementary.pdf]

Supplementary Materials

Discovery of a metabolic signature predisposing high risk patients with mild cognitive impairment to converting to Alzheimer's disease

Yi-Long Huang, Chao-Hsiung Lin, Tsung-Hsien Tsai, Chen-Hua Huang, Jie-Ling Li, Liang-Kung Chen, Chun-Hsien Li, Ting-Fen Tsai, and Pei-Ning Wang

Method S1

Feature-wise normalization using pQC among batches

For the calibration purpose, one pQC sample was selected as a reference among all pQC samples analyzed in different batches by Progenesis QI (Nonlinear Dynamics). The batch-specific correction factor was calculated by equation 1:

$$R_{i,n} = Value_{i,pQCn} / Value_{i, pQCref} \qquad (1)$$

Where $R_{i,n}$ is a correction factor derived from the ratio of feature i value in the nth batch pQC relative to its counterpart in pQC reference. The correction factor was calculated for individual feature independently, then the intensity drift was normalized by dividing the original intensity value of each feature to the correction factor in test samples, as shown in equation 2:

$$Corrected\ Value_{i,n}=Value_{i,n} / R_{i,n} \qquad (2)$$

Where corrected $value_{i,n}$ is the normalized abundance of feature i in samples of nth batch. Drift correction per feature was performed only if the corresponding correction factor is not equal to zero. Finally, the uncorrected features were removed and this method is limited to common features between all batches of pQC.

Figure S1

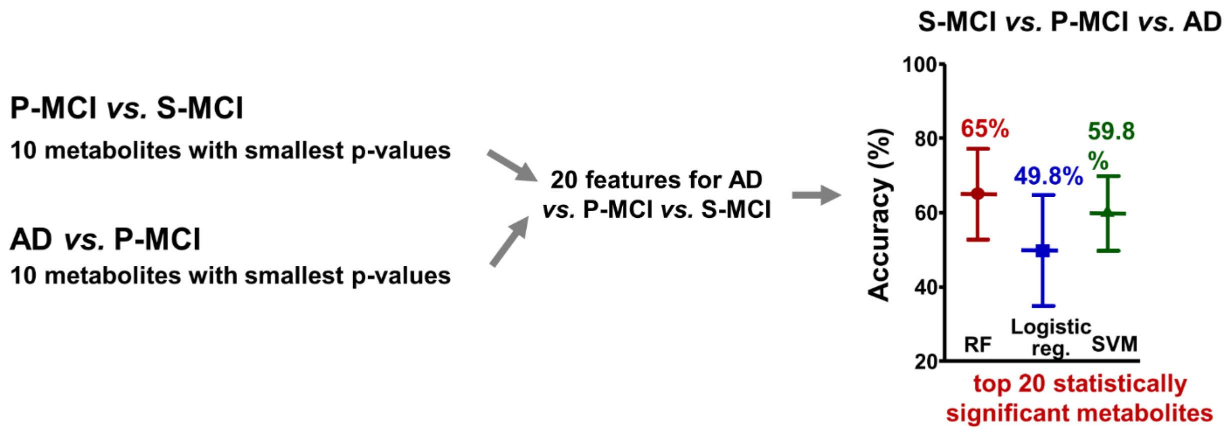

Figure S1. Prediction accuracies of three-class classification models by metabolic features with univariate statistical significance. The accuracies (shown as mean ± standard deviation) were determined using 5-fold cross-validation with 10 repetitions. S-MCI, stable MCI; P-MCI, MCI proceeding to AD.
